# Case Report: Amplified psychoanalysis? Psychoanalysis, OCD and MDMA in a clinical case study

**DOI:** 10.3389/fpsyg.2026.1617428

**Published:** 2026-03-11

**Authors:** Filippo Dellanoce

**Affiliations:** Laboratoire Interdisciplinaire Récits Cultures Et Sociétés (LIRCES EA 3159), Member SIMEPSI (Italian Society for Psychedelic Medicine), Université Nice-Sophia Antipolis, Nice, France

**Keywords:** amplified psychoanalysis, MDMA-assisted therapy, obsessions, OCD, psychoanalytic single-case clinical narrative

## Abstract

This article investigates the novel therapeutic approach of “amplified psychoanalysis” through a detailed examination of the Ygg case, which offers a descriptive single-case illustration of the integration of MDMA-assisted therapy with traditional psychoanalytic treatment for obsessive-compulsive disorder (OCD). The study explores how subjective experience induced by MDMA can interact with an ongoing analysis, potentially enhancing psychoanalytic processes by facilitating access to unconscious material and helping to move beyond therapeutic impasses. The work is presented as a psychoanalytic single-case clinical narrative, acknowledging the value of such cases for generating nuanced insights into psychological phenomena rather than for demonstrating efficacy. In this case, the integration of MDMA experiences within an established psychoanalytic framework appeared to create specific therapeutic opportunities: the altered states of consciousness were experienced by the patient as allowing more direct access to, and processing of, previously avoided memories and affects. This combination may be particularly promising for OCD, where traditional approaches often face limitations; however, its efficacy requires systematic investigation beyond this psychoanalytic single-case clinical narrative. The clinical process analysis highlights several putative processes of change, including enhanced emotional processing, a strengthened therapeutic alliance, and improved access to traumatic memories. The study aims to contribute to both psychedelic therapy and psychoanalytic practice by offering a novel therapeutic perspective on the treatment of OCD. It suggests that “amplified psychoanalysis” may represent a promising direction for future research with larger samples and formal outcome measures.

## 1 Introduction^1^

This article presents a unique psychoanalytic case report: the 9-year analysis of “Ygg,” a man in his thirties, followed through to termination. It reconstructs the main turning points of a treatment marked by interruptions and changes of analyst and highlights the specific challenges of working psychoanalytically with obsessional neurosis (obsessive-compulsive disorder). It also examines how this long-term analytic process intersected with two MDMA-assisted psychotherapy sessions conducted in an underground setting, exploring how the empathogenic effects of MDMA reverberated within, and were in turn shaped by, the clinical context.

The presentation begins with an examination of obsessive neurosis or obsessive-compulsive disorder (OCD) from a psychoanalytic perspective. This is followed by an account of the psychopharmacological properties of MDMA, after which the stages of Ygg’s psychoanalytic treatment are outlined, including descriptions of the two MDMA-assisted sessions. Finally, the notion of “amplified psychoanalysis” is explored.

## 2 Psychoanalysis and OCD

Obsessive-compulsive disorder (OCD) is characterized by recurring, unwanted thoughts, ideas, or sensations (obsessions) that drive individuals to perform repetitive actions (compulsions) to alleviate these thoughts and anxiety. Repetitive behaviors, such as hand washing/cleaning, checking things, and mental acts such as counting or other activities, can significantly interfere with a person’s daily activities and social interactions (American Psychiatric Association [APA], 2022).

S. Freud grappled with the specific symptoms of obsessional neurosis and the difficulties associated with its analytical treatment. His *Rat Man* (1909) remains a reference text for psychoanalysts, describing the core principles of obsessional neurosis. According to Freud, obsessional neurosis is the result of a regression of libido to a pregenital stage, a stage characterized by anal eroticism, in which pleasure and hatred are mixed, and the control of the sphincters and drives assumes a central organizing role. Unfortunately, since this paper, the psychoanalytic literature has produced very few contributions for the treatment of obsessional neurosis (Cooper, 2000; Esman, 2001, 1989; Freud, 1966; Kempke and Luyten, 2007).

Furthermore, the link between obsessional neurosis and OCD remains controversial in psychoanalysis. Several authors have equated the two psychopathological frameworks (Gabbard, 2014; Henrique, 2014; Lima and Rudge, 2015), while others have adopted a more nuanced position.

N. McWilliams distinguishes between ego dystonic obsessive-compulsive symptoms, which are characterized by intrusive thoughts and compulsions that are experienced as alien and distressing, and ego syntonic obsessive-compulsive personality organization, which is characterized by orderliness, perfectionism, intellectualization, and defenses such as isolation of affect and undoing. In this theoretical framework, obsessive-compulsive neurosis is conceptualized as the clinical manifestation of an underlying obsessional personality, which gives rise to ego-alien obsessions and compulsions. Therefore, neurosis is located at the interface: the personality provides the structural background, while the disorder supplies the acute, distressing symptom expression (McWilliams, 2011).

In Freyman’s reading of Freud, an obsession is, first and foremost, an intrusive idea that carries an intense, often unconscious, affect (guilt, remorse, anger). The manifest obsessional thought (“I am contaminated”) is a substitute for a more primal, usually sexual or aggressive impulse (e.g., secret masturbation, infidelity). The compulsive act (e.g., endless hand-washing) is then a second substitute: a protective ritual that attempts to neutralize the underlying guilt. Thus, obsessional neurosis is not simply identical to OCD; it designates a whole chain from personality organization and unconscious conflict, through substituted obsessional ideas, to ritualized defenses. This sequential chain links obsessional character (OCPD-like) with overt OCD-type symptoms (Freymann, 2018).

While Freud established the clinical phenomenology of obsessional neurosis (focusing on anal-sadistic drives, doubt, and mechanisms of defense such as displacement), J. Lacan reinterpreted these phenomena through a structural and linguistic lens. For Lacan, obsessional neurosis is not merely a collection of symptoms but a specific mode of the subject’s relationship to the desire of the Other. Lacan strictly differentiates between the structures of hysteria and obsession based on their relationship to desire; while the hysteric sustains desire by keeping it unsatisfied, the obsessive attempts to destroy the desire of the Other. The obsessive person organizes their world to make desire impossible. He does this because the desire of the Other introduces an intolerable lack of certainty. By destroying or neutralizing the Other’s desire, he attempts to alleviate the anxiety caused by the Other’s castration. To escape the enigma of desire, the obsessive reduces desire to a “demand.” He focuses on the “object of the demand” (often the anal object) because demand is explicit and controllable, whereas desire is elusive (Lacan, 1958; Recalcati, 2012).

Despite theoretical differences, the psychoanalytic literature generally agrees that patients with obsessive disorders are often considered difficult or even “impossible” to analyze. It is also thought that these patients require extended analyses, with sessions occurring multiple times per week (Castel, 2016; Salzman, 1983; Willick, 1995). However, for a considerable number of patients, such high-frequency, long-term treatments are either impractical or financially unfeasible. In response to the limitations of the classical psychoanalytic framework, several authors have argued for the development of more “integrated” or “complementary” approaches. In these approaches, psychoanalytic understanding is combined with other modalities to conceptualize and treat OCD more effectively (Chlebowski and Gregory, 2009; Gauthier, 1999; King, 2017; Woon et al., 2017).

The challenges experienced by patients are not limited to psychoanalytic treatments. Despite the implementation of optimized pharmacotherapy and cognitive-behavioral interventions, a considerable proportion of patients persist in experiencing substantial symptoms and functional impairment (Fineberg and Robbins, 2021). Moreover, up to a quarter of patients may discontinue CBT prior to its completion (Lewin et al., 2005). In light of the limited efficacy and substantial treatment demands, the therapeutic potential of classic psychedelic substances has once again captured the scientific community’s attention. The extant literature describes the use of LSD in conjunction with psychotherapy for the management of obsessive–compulsive symptoms (see Grof, 1980, 2019; Eisner and Cohen, 1958; Johnsen, 1964), as well as more recent work with psilocybin (see Buot et al., 2023; Kelmendi et al., 2022; Moreno et al., 2006; Oded, 2022; Rodrigues et al., 2022) and ketamine (see Rodriguez et al., 2018) in the management of OCD.

## 3 MDMA

3,4-Methylenedioxymethamphetamine (MDMA), commonly referred to as “Ecstasy,” is a synthetic psychoactive substance with stimulant and empathogenic properties. MDMA is known for its ability to enhance mood, increase feelings of emotional closeness, and promote social bonding (Bedi et al., 2009). Originally developed in 1912 by the pharmaceutical company Merck, MDMA was initially used in psychotherapy to facilitate communication and emotional expression among patients (Stolaroff, 2004). However, it was classified as a Schedule I controlled substance in the United States in 1985 because of concerns about its potential for abuse and lack of accepted medical use at that time (Passie, 2018).

The neurophysiological effects of MDMA are complex and involve multiple neurotransmitter systems (Gudelsky and Yamamoto, 2008). MDMA primarily increases the release of serotonin, a neurotransmitter that plays a crucial role in mood regulation, emotional processing, and social behavior. However, MDMA also increases the release of dopamine, which is associated with rewards and pleasure, and norepinephrine, which affects arousal and alertness (Parrott, 2013). MDMA also induces oxytocin release (Kirkpatrick et al., 2014).

The psychological effects of MDMA are diverse and can vary significantly depending on the dose, individual’s psychological state, and context in which it is used (Baggott et al., 2015; Bershad et al., 2016). MDMA users often report intense feelings of happiness and wellbeing, commonly referred to as a “high.” MDMA is known for its entactogenic effects, which promote feelings of emotional closeness, trust, and empathy toward others, and many users experience enhanced sociability and a desire to engage with others (Dumont and Verkes, 2006; Dumont et al., 2009). MDMA can cause changes in the perception of time, making it feel as though time is passing more slowly or quickly. Users may also experience enhanced sensory perceptions, such as an increased appreciation for music, colors, and tactile sensations (Fox et al., 2002). MDMA has also been shown to decrease anxiety and fear (Luoma and Lear, 2021; Wolfson et al., 2020). Users often report an increase in cognitive and emotional empathy, allowing them to better understand and relate to others’ feelings (Schmid et al., 2014)^2^.

3,4-Methylenedioxymethamphetamine-assisted therapy (MDMA-AT) is a therapeutic approach that combines the administration of MDMA with psychotherapy in a controlled and supportive environment. This method is designed to enhance the therapeutic process, particularly for individuals dealing with trauma-related conditions, such as post-traumatic stress disorder (PTSD). In this approach, MDMA is administered in a clinical setting under the supervision of trained professionals. The dosage is carefully controlled, and the sessions are structured to ensure safety and efficacy. Therapists provide support, guidance, and validation, which can enhance the patient’s sense of safety and trust during the experience (Feduccia and Mithoefer, 2018; Ot’alora et al., 2018). After the MDMA session, therapists work with patients to integrate the insights and experiences gained during the session into their everyday lives. This integration process is crucial for translating the therapeutic benefits of the MDMA experience into lasting changes in behavior and emotional wellbeing of the patients. MDMA-AT is supported by a growing body of research demonstrating its efficacy in treating PTSD and other clinical conditions (Sessa et al., 2019). Clinical trials have shown significant reductions in symptoms and improvements in overall wellbeing for participants undergoing this treatment, mostly for PTSD (Mitchell et al., 2021, 2023).

3,4-Methylenedioxymethamphetamine facilitates therapeutic processes through several mechanisms that enhance emotional processing, interpersonal connections, and the overall therapeutic experience (Wardle and de Wit, 2014). MDMA has anxiolytic properties that can help patients confront traumatic memories and experiences without becoming overwhelmed by fear or anxiety (Mithoefer et al., 2010). This allows individuals to engage more fully in the therapeutic process and explore their difficult emotions (Bahji et al., 2020). The drug promotes feelings of empathy, trust, and emotional connection, which can strengthen the therapeutic alliance between the patient and therapist (Borissova et al., 2021). This increased sense of connection can lead to more effective communication and a greater willingness to explore personal issues (Krediet et al., 2020). Patients often report feeling more open and willing to share their thoughts and feelings, facilitating deeper therapeutic engagement (Tedesco et al., 2021). MDMA may enhance the recall of traumatic memories while reducing the emotional distress typically associated with these memories. This allows patients to process and integrate their experiences in a therapeutic setting without becoming overwhelmed by negative emotions (Mithoefer et al., 2013). The drug induces feelings of euphoria and wellbeing, which can create a positive therapeutic environment. This positive emotional state can help patients approach difficult topics with a sense of safety and support, making it easier for them to engage in challenging therapeutic work (Tedesco et al., 2021). MDMA affects neurotransmitter systems, particularly serotonin and oxytocin, which are involved in mood regulation, emotional bonding, and social behaviors. The increase in oxytocin levels may contribute to feelings of trust and connection, further enhancing the therapeutic experience (Oeri, 2021). MDMA can enhance the effectiveness of various therapeutic techniques such as imaginal exposure and cognitive restructuring. Although Stanford University has recently proposed a clinical trial combining CBT with MDMA for the treatment of OCD^3^, there is currently no empirical evidence supporting the use of MDMA for OCD in the literature. It is precisely at this juncture that Ygg’s case may contribute to the emerging discussion on the use of MDMA-AT for OCD.

## 4 Methods

The present article is a psychoanalytic single-case clinical narrative grounded in routine analytic practice. It is best understood as a psychoanalytic clinical case report rather than as a qualitative case study in the empirical sense. The primary objective is to describe and theorize the process dynamics related to what is proposed as “amplified psychoanalysis,” rather than to provide proof of efficacy or a standardized evaluation of outcomes. In this article, “amplified psychoanalysis” refers to an integrative approach in which non-ordinary states of consciousness – induced, for example, by MDMA or classic psychedelics such as LSD, psilocybin or DMT – are deliberately embedded within an ongoing analytic treatment in order to potentiate psychoanalytic processing and to facilitate the emergence and working-through of unconscious material.

No formal ethics committee approval was obtained for this retrospective single-case report based on routine psychoanalytic practice. In the author’s institutional context, retrospective single-case reports based on anonymized clinical material collected in routine care are exempt from formal ethics committee review; accordingly, no formal ethics approval was sought for this case. The patient provided written informed consent for the publication of his anonymized clinical material, including his accounts of MDMA experiences undertaken within a therapeutic context. All potentially identifying details have been modified or omitted to safeguard confidentiality.

At the time of the MDMA sessions, MDMA was a controlled substance in France, and its therapeutic use was permitted only within authorized clinical trials. The two MDMA sessions described here occurred outside such a regulated framework, in an already existing non-regulated (“underground”) therapeutic setting that the patient consciously and autonomously chose to attend. The author did not organize, prescribe, or administer MDMA and had no role in the medical management of those sessions. This report documents the clinical material that subsequently emerged in psychoanalytic treatment and discusses its clinical, theoretical and research implications. The manuscript does not advocate or recommend the unregulated use of MDMA, and any possible future development of “amplified psychoanalysis” is explicitly envisaged only within legally regulated, ethics-approved protocols.

The patient, hereafter referred to as “Ygg,” was a male in his thirties at the time of the MDMA sessions. He was employed, in a romantic relationship, and had received university education. A pre-existing diagnosis of obsessive-compulsive disorder (OCD) was made by two psychiatrists (one private and one from a hospital). The author did not administer a structured diagnostic interview or a standardized OCD severity scale, as this is not standard practice in psychoanalytic clinics, at least in France. The diagnostic framework was thus based on the documented psychiatric diagnoses and clinical phenomenology reported in the treatment. The presentation of the primary symptoms included two main categories: obsessive thoughts centered on the fear of harm to a loved one (for instance, there could be a concern that the partner’s smoking, alcohol consumption, or drug use could result in death or separation) and compulsions that manifested as overt checking or verification rituals, as well as covert anxious rumination, in order to prevent or neutralize obsessions and related unpleasant emotions (anxiety, fear, panic). During periods of heightened distress, the patient exhibited symptoms such as panic attacks, insomnia, challenges in academic and occupational functioning, episodes of withdrawal, and a tendency to refrain from engaging in romantic and social interactions. The clinical severity was pragmatically inferred from the patient’s need for pharmacological support (previous treatment with SSRIs for approximately 1.5 years and an anxiolytic available as needed) and multiple previous therapeutic attempts with limited benefits. Prior to the commencement of the present analysis, the patient had already accumulated a substantial psychoanalytic history, spanning approximately 6 years, marked by changes in analysts and periods of interruption. Previous interventions included 1 year of cognitive behavioral therapy (CBT) in a hospital setting with limited effects, mindfulness-based stress reduction (MBSR protocol), and approximately 1 year of metacognitive therapy (MCT). At the time of the MDMA sessions, the patient had discontinued SSRI therapy; however, alprazolam remained available as needed.

Psychoanalytic work was conducted on a couch. The frequency of sessions was twice weekly during a previous analytical phase and once weekly with the author of this study.

The patient underwent two MDMA-assisted sessions in France in an underground therapeutic setting. The author did not participate in these sessions and did not interview the MDMA therapists; the information reported below is based on the patient’s reports, integrated into the subsequent psychoanalytic work, and the author’s clinical notes. Each MDMA session was preceded by preparatory meetings with a pair of therapists and followed by an integration meeting. During the MDMA sessions, the patient reported the uninterrupted presence of the therapists, the incorporation of music, and the use of eye-masks. During the initial session in September 2021, the patient reported ingesting 125 mg of MDMA, with an additional 50 mg administered later in the session; he described the experience as lasting approximately five and a half hours. During the second session in March 2022, the patient reported ingesting approximately 150 mg MDMA, followed by a supplemental 50 mg later in the session, which lasted approximately 6 h. The veracity of the reported safety and monitoring information could not be independently verified. Physiological safety during the MDMA sessions was not monitored with standardized measures (e.g., blood pressure, heart rate); apart from the patient’s continuous self-report, no objective vital-sign monitoring data were available to the author. The only adverse effects reported by the patient were temporary jaw clenching and mild headache after the session. No serious adverse events were observed or reported.

This case study, together with the accompanying written reflection, draws on the following sources. First, contemporaneous notes from psychoanalytic sessions were taken during and/or immediately after the sessions: these included the patient’s narratives of the MDMA experiences, which were subsequently elaborated upon in the analysis. Second, after-session (*après-coup*) notes were written, including countertransference experiences, hypotheses, and process formulations. Third, reflections from clinical supervision are drawn upon, with the focus being on psychoanalytic treatment rather than on the MDMA sessions themselves. No audio or video recordings were collected, nor were structured interviews or standardized symptom rating scales used. No collateral data were obtained from the MDMA therapists. The analytic approach consisted of a clinical process analysis in the psychoanalytic tradition. After completing treatment, the author repeatedly reviewed the clinical notes and follow-up material, identified clinically salient “turning points” (e.g., shifts in obsessive symptoms, changes in affect tolerance, and modifications in transference configurations), and grouped these into a small number of overarching themes that structure the Discussion (emotional processing, transference/analytic field, and trauma memory). No formal qualitative coding scheme or inter-rater analysis was used, in line with psychoanalytic single-case methodology, which treats theory-guided clinical interpretation as an integral part of the research process. From a reflexive standpoint, the author’s dual role as Ygg’s analyst and as interpreter of the case is acknowledged as both a limitation and a defining feature of this method; to enhance reflexivity and minimize blind spots, the material was regularly discussed in clinical supervision.

Symptomatic changes and functional outcomes were described qualitatively based on the evolution of the analytic material, the patient’s self-reports in weekly sessions, and a planned follow-up meeting 18 months after termination. No standardized OCD rating scales, structured follow-up interviews, or independent clinician ratings were employed. Consequently, references to “remission” or “recovery” in this article refer to a clinically observed remission, as perceived by the patient and the treating analyst, rather than a formally quantified treatment effect. The material was selected to highlight clinically relevant turning points related to the proposed concept of “amplification,” including changes in affective tolerance, associative range, defensive organization, and transference/countertransference configurations. Particular emphasis was placed on the subsequent processing of experiences with MDMA in psychoanalytic sessions, with integration conceptualized as a process that unfolds over time within the analytical framework. In accordance with psychoanalytic principles, the representational and affective content that emerged during MDMA sessions was treated as if it were dream material.

## 5 Ygg: clinical history and therapeutic failures

Ygg^4^ commenced his analysis at the age of 22 to resolve the anxiety he experienced when he was obliged to return home every weekend to his family for Sunday lunch. This initial treatment enabled him to explore his relationship with his father, who had left home when Ygg was 12, and to explore fears linked to his homosexuality and, above all, anxieties linked to his romantic life and his then-partner. Anxieties arising from this romantic relationship reached an intolerable level, and analytical treatment proved ineffective in addressing the underlying issues. In 2015, Ygg opted to depart from his familial, romantic, and social networks, relocating to France, where he promptly sought the guidance of a new psychoanalyst, in particular a Lacanian specialist.

In accordance with the tenets of Lacanian psychoanalysis, the neurotic subject commences analysis proper at a designated juncture following a requisite period of time referred to as the “preliminary interviews” (Freymann, 2016). The subject will only enter the analysis once the analyst authorizes it on the basis of established transference, a moment that may be accompanied by, but is not defined by, the use of the couch. During preliminary interviews, which typically last several months, subjects present their complaints, suffering, and dissatisfaction with certain aspects of their lives, which they begin to explore. From a more general psychotherapeutic perspective, this preliminary work can be understood as an extended assessment and engagement phase in which symptom-focused help-seeking gradually shifts toward a reflective exploration of the meanings and relational contexts of the symptoms. However, at a certain point, because of the analyst’s interventions and the establishment of a transferential relationship, there is a transformation in the subject’s behavior, particularly in the way they speak and engage with their symptoms and suffering. A desire to know (*désir de savoir*, in French) *may* emerge, prompting the subject to seek explanations for their suffering elsewhere in the unconscious. The culmination of the preliminary interviews is typically marked by the formulation of an “analytic demand” (*demande analytique*), which is a “quest” for knowledge and an opening to the unconscious.

In Ygg’s case, this quest to know took the form of the question, “*How do I go about leaving?*” This question arose at the conclusion of a series of associations linked to a dream in which, upon leaving the therapist’s office, the subject stumbles near the doorway.This “analytic demand” is a “demand for knowledge” (*demande de savoir*) (Izcovich, 2017; Lacan, 2001; Miller, 2017). In more general psychodynamic terms, this corresponds to the development of an observing position and motivation to understand, rather than simply eliminate symptoms. Additionally, Ygg spontaneously articulated this question using elements of both Italian (*come fare* – “How do I do”) and French (*pour partir?-“to* leave”). This demand remained enigmatic for both the subject and the analyst, unfolding within a field of meaning that profoundly shaped the subject’s life. The signifier *leaving* carried multiple possible meanings, including departing from one’s country, mortality, travel, the dissolution of a relationship, and even the cessation of Sunday lunch traditions. At the same time, it encompassed other meanings that would be progressively elucidated through Ygg’s dreams and symptoms throughout this analysis.

During the analysis, Ygg became aware of and worked through the obsessive-compulsive behaviors and rituals that had troubled him in the past. One particularly noteworthy memory to which he repeatedly returned is the “souvenir of the corridor”: following a second relocation in 2011, he temporarily resided with his mother and brother at his maternal grandparents’ home, whom he held in high regard. On the evening in question, he retired to his bedroom and sought solace in solitude. As he proceeded down the corridor, his thoughts turned to the possibility of his grandmother’s death, compelling him to pass by his grandparents’ bedroom-located on the left side of the corridor-at which point he became immobilized by distress, unable to move. These intrusive thoughts and the accompanying anxiety persisted for an extended period, during which he was unable to cease ruminating on them. From this memory, Ygg was able to retrieve many of his obsessive concerns, particularly those involving his parents or partner. For example, he experienced anxiety when he observed his partner engaging in lengthy telephone conversations without the use of headphones, fearing that exposure might lead to brain cancer. To manage this fear, he purchased a dozen headphones for him. He also tended to overreact when his plans failed. It was precisely in search of a solution to his anxiety that Ygg chose to leave his country in 2015. He left to avoid the possibility of seeing his parents or partner “leaving,” whether as a result of a perceived threat or for some other reason.

At this juncture, transference became increasingly challenging, with the analyst now finding herself affected by Ygg’s doubts and obsessions. Ygg awoke each day contemplating the possibility of seeking counsel from another therapist, given the lack of benefit he felt he had derived from his current course of analysis. At this stage, Ygg’s predominant feelings toward the analyst were anger and hatred. In one final dream, Ygg reports having been “had” (*si è fatto fregare*, in Italian)^5^ by the analyst. This dream is followed by the recollection of a childhood memory. After moving house for the first time at the age of seven, Ygg recalls a scenario of anxiety and fear that would impose itself on his mind every night before he went to sleep.

“A film of thoughts ran through my mind: the bogeyman would enter the room where my little cousin, a baby, was sleeping; he would take her from her cradle and slam her against the wall, holding her feet. I was distressed and on my knees. To dislodge this horrible image, I began counting, praying, and thinking about God or beautiful images in bed.”

This was potentially the first occurrence of this symptom. At the age of seven, Ygg experienced two significant events: the family’s relocation to a new residence – which represents a significant transition in Ygg’s psychic life – and the birth of his cousin. Furthermore, it is worth noting that each obsessive symptomatic manifestation exhibits a profound structural analogy to the childhood obsession with the bogeyman: in later fears concerning cancer, illness, or death, the threat is always directed toward a beloved other – his parents or his partner-and never toward Ygg himself. Initially, Ygg attempts to alleviate the anxiety through overt rituals (such as purchasing headphones) and covert rituals (including praying to gods or thought suppression) (Purdon, 2008). When the anxiety becomes unbearable and these rituals are no longer able to contain it, leaving becomes his only perceived solution – whether by going abroad or via terminating a relationship. In each case, leaving functions as a way of distancing himself from the object of obsession in order to avoid the distress caused by its presence^6^.

Over time, the situation deteriorated, with Ygg’s obsessions shifting from one partner to another.

In the case of one partner – a young man who does not smoke regularly – it is the cigarette that becomes the perceived source of threat. If the partner smoked during an evening, the resulting anxiety quickly escalated, accompanied by an intense sense of alertness and danger. Ygg began to experience panic attacks following his partner’s announcement of plans to attend a social gathering where, according to Ygg, smoking would occur. He is preoccupied with ruminating on the party announcement and potential scenarios involving cigarettes. To circumvent the onset of anxiety, Ygg endeavors to establish novel rituals in the context of a subsequent relationship with a partner who smokes regularly. One such ritual involves Ygg’s avoidance of searching for traces of cigarettes in his partner’s apartment. Upon noticing an ashtray on the balcony, he deliberately averted his gaze and swiftly closed the curtain, citing an unrelated reason for doing so. Similarly, if his partner’s clothes exhibit a smoky odor, he refrains from kissing him to avoid the smell. Paradoxically, the more these rituals manage to deal with anguish in waking life, the more the anguish returns at night, in dreams. He would awaken in a state of anxiety when, within the dreamscape, he discovers cigarettes within a box of pasta or a full ashtray in place of a glass of water. Each dream would end abruptly at the precise moment that Ygg identified the object perceived as threatening, upon which he awoke experiencing anxiety, alertness, and exhaustion.

With this last partner, the situation began to reach a critical point. Ygg’s ruminations and obsessions persisted throughout the analysis sessions, effectively transforming the sessions into extended rumination on his condition and obsessions. Despite 4 years of analysis with the second analyst, Ygg remained unable to effectively manage his symptoms. Faced with what he experienced as the analyst’s unresponsive and unhelpful silence, Ygg sought consultation from psychiatrists, who ultimately confirmed a diagnosis of OCD.

Following his departure from the second analyst, Ygg commenced a course of pharmacological treatment with sertraline (150 mg) for 1 year, in conjunction with cognitive behavioral therapy (CBT) and mindfulness-based stress reduction (MBSR). Subsequently, he underwent metacognitive psychotherapy (MCT) using the approach outlined by Fisher and Wells (2008), Wells (2011). Alprazolam (0.25 mg) was prescribed on an as needed basis to assist with sleep. By the conclusion of this course of MCT psychotherapy, Ygg reported a cessation of panic attacks in previously triggering situations, as well as an improvement in sleep quality; however, his symptoms had not yet fully resolved.

At the end of this period, Ygg contacted me to resume analysis, prompted by a dream involving a “black-veiled woman,” which he described as follows:

“In a flat, there’s a wall at right angles to me: behind the wall is a black-veiled woman I do not recognize. The only aspect of the woman’s face that I can discern is her eyes. Her expression is malevolent and sarcastic. I am overcome with a profound sense of animosity toward her, a feeling of intense anger and hatred. In front of the wall is my partner, and next to him, in the air, scroll all the names of objects that could potentially pose a threat to him (cigarettes, cancer, drugs, etc.). All I feel toward him is panic: *I have got to do something!*”

In this dream, the black-veiled woman assumes the role of the bogeyman, while Ygg’s anxiety is once again directed toward a beloved object perceived as being in danger. His hatred finally emerges in accordance with Freud’s theory of obsessional neurosis (1909). The dream is also marked by a significant moment of self-reflection. Ygg asks himself, “does not this represent my own behavior with my partners? I intervene when I am distressed, for example, to prevent them from smoking.” Additionally, he associates this dream with two earlier memories in which he experienced such intense hatred and rage that his arms were rendered partially paralyzed. In both instances, the objects of his hatred were women who, he felt, had attempted to “have” him again.

## 6 Sessions with MDMA

(a) The first session (September 2021)

By the end of the initial year of analysis, in September 2021, searching for alternative and definitive options, Ygg had the opportunity to meet a couple of therapists who conduct underground sessions of empathogenic and psychedelic substance-assisted therapy^7^. Since the prohibition years, informal communities of therapists have continued to operate underground, accompanying clients during sessions of substance-assisted therapy (Glynos et al., 2024). These practices follow, to varying degrees of fidelity, the knowledge and practices that were formalized in the early days of psychedelic-assisted psychotherapy (Adamson, 2012; Grof, 1980; Stolaroff, 2004).

Following ingestion, the effects of MDMA became evident. Two broad categories of images predominate in Ygg’s consciousness. The first consisted of autobiographical memories of his life, largely organized around experiences of separation, deaths (including the demise of a cat, two family friends, and his grandfather), and challenging episodes from his childhood and adolescence (including separation of his parents, relocating, and so forth). The second category comprised mental images that appeared as unfamiliar or novel “past memories,” insofar as they did not align with Ygg’s known autobiographical history. These images carried the subjective quality of recollected dreams. When viewed in sequence, they collectively convey the following narrative:

“Children in uniform are playing in the distance. I am crying because I am not playing with them. My entire body is crying. Someone told me: “If you want to be a good boy, you do not have to play like other children.” I’m going to leave. I’m sad, I’m alone. I am on the verge of something. I threw myself into the water without any thought. Have I been found? They should have been looking for me.”

In the concluding phase of the experience with MDMA, Ygg encounters a bogeyman in a corridor. Approaching him, Ygg states, “Your presence is now futile as there are no longer any children. You are free to depart now.”

The session seems to have enabled a “figuration,” in the sense of dream work and Freudian *Darstellbarkeit* (Freud, 1900), of separation in its most extreme form, that of death. The image stages a departure from the other children, who remain happily playing in the distance, and renders this separation available to be explored. At the same time, two elements emerge that are new in relation to Ygg’s previously stereotypical reasons for ending relationships – and to what the analytic work had thus far allowed him to understand. First, Ygg leaves not in response to danger or threat, but because “good children do not play like others,” (they do not smoke, they do not drink, they must avoid getting sick). That is, Ygg leaves in response to an internal injunction, a reproach against a lifestyle that he has learned to regard as reprehensible. This movement can be understood as a revelation of the superego, or as the introjection of rigid familial moral interdictions. More importantly, the final sentence, “They should have been looking for me,” reveals Ygg’s deep desire – that they would search for him, that they would take an interest in him. At the same time, it foreshadows the hatred for not doing so, which will become revealed in the following session.

In the subsequent morning integration session, Ygg was able to fully engage with the overwhelming sadness associated with this separation. He was finally able to experience release from the pervasive sadness that had accompanied him in the past when he felt compelled to leave those he loved in search of relief from his anxieties. In a display of profound emotion, he wept openly in the presence of therapists, marking the inauguration of the integration of the material that had emerged during the non-ordinary state of consciousness (Bathje et al., 2022).

Ygg’s sorrow persisted for several weeks thereafter. During this period, he discussed his experience with me and was able to revisit each sad memory encountered during the session individually, with a gradual attenuation of their emotional impact. I determined that the most appropriate course of action would be to treat this account – together with all the associations that Ygg produced – as a dream narrative, the two phenomena having similar neurophenomenological and psychological structures (Carhart-Harris and Friston, 2010; Koslowski et al., 2023). Subsequently, Ygg’s discourse and manner of expression exhibited notable changes: they became calmer, more attentive, and slower, suggesting that the memories of the experience continued to evoke a sense of awe. At the same time, he demonstrated the ability to experience a broader range of emotions, including joy and laughter, within the same session. This shift in emotional expression was accompanied by greater logical coherence in his speech, suggesting a progressive transformation in his relationship to the material that had emerged during the experience. Additionally, a sense of continuity emerged across sessions, with several instances in which the narrative seemed to resume from the previous session’s endpoint. I was struck by the gravity of these sessions, which imbued the setting with a profound seriousness and noticeably altered the subjective experience of time and space. My interpretation was not required. Ygg advanced through the work recalling memories, forging connections between ideas, independently elaborating their meanings, and incorporating new material into each session. As this was my inaugural experience of this phenomenon, I deliberately confined myself to the role of an observer, thereby ensuring a space capable of containing the psychic material rendered accessible by the MDMA experience (Bion, 1963, 1962). As Barrett notes, drawing on the work of Bollas (2012), when the internal experience itself becomes the driving force of the treatment, interpretation is no longer necessary and, instead, the analyst assumes the position of a witness (Barrett, 2022).

Ygg had two additional integration sessions with the therapist couple. In parallel, and in an asynchronous manner, he was able to continue the analysis work with me, a process that was clearly facilitated by his MDMA experience. The analysis sessions appeared to build upon and elaborate the work initiated during the MDMA experience itself. In summary, the MDMA experience appeared to reduce the rigidity of the ego’s defensive mechanisms, while facilitating retrieval of previously repressed material (Buchborn et al., 2023).

The bogeyman was frequently referenced during the sessions. Ygg recalled that, in his culture, a lullaby exists that may be translated as follows: “*Lullaby, oh lullaby/ This baby I’m going to give it to / I’m going to give it to the old lady / So that she can keep it for a week / I’m going to give it to the bogeyman / So that he can keep it for a whole year…*”. The lullaby, in its original version, culminates in a decisive manner, whereby the infant is not ultimately entrusted to the bogeyman. However, in Ygg’s memory, the narrative remains unresolved. He recalls one occasion, at the age of seven, during a family dinner: his mother was holding his infant cousin in her arms and singing the lullaby to her, repeating it each time after the bogeyman’s appearance. Ygg recalls listening intently, waiting for the song to continue beyond this point, hoping for a different concluding phrase. The incident occurred before the family meal. Ygg was seated next to his grandmother. When his mother began singing, he found himself unable to discern any other auditory stimuli apart from her voice. Upon later inquiry, it was found that Ygg’s mother was unable to recall the lyrics which followed mention of the bogeyman in the song. With respect to the bogeyman, we cannot fail to note that, in the images emerging under MDMA, the bogeyman appears in the corridor. His phantasmatic torture of the little cousin takes place in the room located at the far right of the corridor in the grandparents’ house. In that same corridor, at the far left, Ygg experiences his first anguish in relation to the death of his grandparents. In the lullaby, the baby is left in the hands of the bogeyman for a month; however, because Ygg never knew the lullaby’s decisive ending, it is as though the baby remained with the bogeyman forever. No one went to look for him – although Ygg, who is also the child dressed in uniform – would have wished to do so. The MDMA session thus appears to have inaugurated a process of de-condensation, which constitutes the opposite of the work of dream and symptom formation.

A few days after the MDMA session, Ygg and his partner were invited to a party. When his partner began smoking, Ygg resisted and left. During the subsequent psychoanalysis session, Ygg noted a marked change in his behavior: “In the past, I would have stayed until the end of the evening to keep an eye on the situation…but not now. I left, mad as hell! He can smoke all he wants, but without me, that bastard!” What emerges here is hatred entering consciousness directly, no longer disguised as anguish in its inverted form (Freud, 1915, 1909).

Furthermore, in subsequent dreams, the image of the partner underwent a transformation, assuming feminine characteristics, thereby paving the way for Ygg to engage in an analysis of the figure of his grandmother. In December 2021, a particularly significant dream, entitled “The Dream of the Jacket,” signaled the continuation of the project. In this dream, Ygg finds himself at a social gathering with his partner and a woman. He then reaches into his jacket pocket and retrieves a packet of cigarettes. He regards his partner with newfound awareness and realizes that both the cigarettes and jacket he is wearing belong to the woman. In the session, he states that neither his anxiety nor his hatred is directed at his partner, but rather toward the woman who appeared in the dream. He elaborates: “The threat comes from the lady. I’m carrying her jacket and her threat. I hate her, and she is waiting for me.”^8^

(b) The second session with MDMA

The second MDMA session begins where the first one concluded-namely in the corridor – but this time in the absence of the bogeyman. The experience then unfolds into an empty house. Although autobiographical memories re-emerge during this session, the dreamlike quality predominates. Ygg begins to recall a romantic relationship between a homosexual man and a woman who is in love with him, a relationship that took place during a past war. The hatred that accompanies Ygg throughout this session is of a magnitude previously unexperienced by him. In this recollection, the woman is referred to as “A,” a name that bears a phonetic resemblance to that of Ygg’s grandmother.

Ygg recounts a narrative composed of elements of perversion, hatred, violence, misunderstanding, disappointment, sexuality, and separation, akin to a highly detailed dream. Once more, he reiterates that this woman “*m’ha fregato*!”^9^ As the session progresses, Ygg encounters a box containing a note. One side of the note reads “fuck me!” while the other side reads “kill me!” Here again, love and hatred are conflated, their boundaries obfuscated as they merge and intertwine. At a certain point in the session, Ygg’s hatred intensifies, giving rise to thoughts of physical violence directed toward both Lady A and his former partner. In response, one of the therapists offers him a pillow, suggesting that he give bodily expression to this hatred and violence. Ygg then begins to produce vocalizations that evoke the rhythmic sounds associated with sexual activity.

Following the session, Ygg reported having the following dream:

“I’m in my grandmother’s kitchen. She is all dressed up with a beautiful pearl necklace. The only thing separating us is the table: I feel like she is following me, trying to catch me. I look into her eyes, and, for the first time, I say to myself that she must have been a big cunt!”

From “A”, the woman “met” in the MDMA trip initially described as a whore, to the grandmother – now evoked in the same register – the figures converge. In this scenario, the table replaces the wall, the grandmother is depicted in place of the black-veiled woman, and the hatred directed toward her becomes unmistakable. Ygg is astonished by this dream, given that his grandmother is the individual he holds in the highest regard and for whom he has the greatest affection for. Approximately two weeks later, an auditory hallucination occurred during a session. Ygg reports: “I heard what sounded like a woman screaming! It was like a foreign memory that belonged to me. She screams in panic and anguish. Who is she? Didn’t you hear her? I am not even sure myself; I am stressed now.” A few sessions later, this hallucination led Ygg to what he would describe as the most important memory of his entire analysis, the “souvenir of the door”: Ygg is 6 years old and it is the morning his mother tells his grandmother – her mother – that they are moving to the neighboring village. The grandmother, standing in front of the kitchen door, swears back: “*You are taking him away from me*,”^10^ referring to Ygg. She raises her hands to her face and begins to cry. This memory was later confirmed by Ygg’s mother, who was present. From this point onward, Ygg begins to analyze the figure of his grandmother, a process accompanied by several dreams and memories that mark the final phase of his analysis.

(c) The last dream

In May 2022, Ygg shared a dream that he had pondered extensively, marking the conclusion not only of an emotionally complex period but of the analysis as a whole. It is the dream of the “*bouquet-de-couverts*” (bouquet of cutlery):

“I am sitting on the bed in my uncle’s room, her last son, in my grandmother’s house. Beside me, my ex-partner sleeps. My grandmother comes down the corridor with a bouquet of cutlery in her hands. She offers it to me like a treasure and a gift, and then she leaves, heading back down the corridor.”

This dream once again presents a triangular structure involving Ygg, his grandmother, and his partner, now with the addition of an object. However, the hatred and anxiety that were present in the previous dream have disappeared. The dream is set in the bedroom of Ygg’s uncle, as was the case in the earlier obsession with the bogeyman. In the bed, Ygg appears to take up the position of his grandmother’s symbolic fourth child, her last son. The bouquet of cutlery now serves to replace the threatening object (cigarettes, drugs, cancer, etc.). The formerly threatened object – whether the little cousin, partner, mother – can now sleep peacefully beside Ygg and is no longer experienced as endangered. The earlier obsession with the bogeyman was situated in the uncle’s room, at the far right end (A); by contrast, the “memory of the corridor” is architecturally anchored near the grandparents’ room, opposite the uncle’s room, at the left end (B); and the “dream of the bouquet of cutlery” thus returns to the initial location (A). This psychic-symbolic movement, involving both the construction and deconstruction of Ygg’s neurosis, appears to be accompanied by a temporal displacement and a countervailing elaborative movement – namely that of the analysis process itself. Anxiety related to loss and separation is thereby eliminated. In this dream, the grandmother arrives from the corridor to present Ygg with a gift. In contrast, in the obsession with the bogeyman, in the dream of the veiled woman, and in other obsessions, an object was taken away, and it fell to Ygg to protect it from the threat posed by the bogeyman.

The cutlery she presents to him consists of the utensils he had previously used to prepare the dining table for weekend meals. At the same time, it also represents the cutlery from his grandparents’ restaurant, an object which he fantasized about throughout his youth. The grandmother thus appears to be saying to him: “I am leaving you my heritage – my cutlery, the cutlery from my restaurant – and it will now be up to you to prepare Sunday meals for those you love.” The dream therefore appears to articulate the transmission of desire and the continuity of the bond, rather than separation.

However, in French, the term *bouquet-de-couverts* (literally, “bouquet of cutlery”) may also be understood in two additional ways: as *bouquet-découvert* (a bouquet that has been discovered), and as *bouquet-de-découvertes* (a bouquet of things that have been discovered). This semantic polysemy resonates with the figure of the child dressed in uniform, who would have wished to be found or discovered. Moreover, in French, “*ajouter un couvert*” (“to add a place setting”) means to add a place at the table: the one who arranges the cutlery also arranges the table, the seating, and assigns each diner to their unique position. In Italian, the equivalent term would be *mazzo di posate*, a bouquet of utensils- objects that have been “placed” (*posati*) throughout the analysis, and which now allow one to eat and enjoy the meal in company. One may also recall the expression, a *mazzo di coperti*: the “*coperto*” in Italy refers to the charge one pays simply to sit at a table in a restaurant – which includes service, cutlery, and bread – marking the symbolic cost of having a place. However, what had previously been covered is now uncovered and brought into view. This image thus stands as a sublime instance of linguistic, imaginal, and semantic condensation work structured around a symbol.

The circle is thus complete: Ygg began his analysis with an anxiety linked to returning to the family home to eat; he subsequently formulated his demand as the question of *How to go about leaving?*. These elements are condensed and resolved in the image of the bouquet of cutlery.

From the period following the final MDMA session until the end of analysis, Ygg reported a progressive attenuation, and then an absence, of obsessions, compulsions, panic attacks, and avoidance rituals in everyday life, and OCD-related phenomena no longer dominated the analytic material. Dreams were less frequently organized around cigarettes, cancer, or other threatening objects, and the sessions became progressively less structured by ruminative processes. At a follow-up visit conducted eighteen months after the conclusion of analysis, he described a stable romantic relationship with a partner who smoked and continued to report no OCD symptoms in daily life. He spontaneously characterized himself as “cured” and “transformed,” and as finally able to live without constant worrying. These observations, however, remain observational and subjective: they are based on the patient’s self-report and the treating analyst’s clinical judgement, and may also reflect the cumulative effects of long-term psychoanalytic work, natural fluctuations in symptom severity, and expectancy factors, in addition to any contribution of the MDMA-assisted sessions. No standardized symptom scales, structured diagnostic reassessment, or independent outcome ratings were obtained.

## 7 Discussion

As is well-documented, Freud left only single case studies, from *Studies on Hysteria* to the celebrated *Five Psychoanalyses*. Since its inception, psychoanalysis has been structured around a culture of the single case (Meganck et al., 2017), with the contributions of M. Klein and D. Winnicott – among the most prominent Figures – continuing to function as central points of reference in the field (Kächele et al., 2012). Although the Ygg case remains a psychoanalytic single-case clinical narrative it would be erroneous to underestimate its validity. Single-case studies continue to provide novel insights into psychological phenomena. As Nickels et al. (2022) have shown, such studies facilitate the identification of individual patterns that might otherwise remain obscured, while offering rich, fine-grained data. Qualitative research, in particular, plays a crucial role in understanding patient experiences and therapeutic processes in psychedelic treatments for mental disorders, helping to elucidate common themes such as insight, altered self-perception, and increased connectedness (Breeksema et al., 2020).

Within this framework, the objective of the present single-case study is not to generalize in a statistical sense, but rather to propose a conceptual model of “amplified psychoanalysis” that may be applicable to other cases of OCD and, more broadly, to neurotic configurations that prove resistant to conventional treatments. Ygg may therefore be considered an index case, insofar as it delineates in detail the conditions, settings, and processes – such as the specificity of OCD symptomatology, the transference history, and the modes of integration between MDMA-AT and analytic work-that can be emulated and examined in future studies, including case series, single-case designs, and controlled pilot trials. In this sense, the present study does not assume that all patients with OCD respond in an identical manner. Rather, it proposes a model of mechanisms of change that can be systematically tested and, if necessary, reformulated using larger sample sizes. The Ygg case also represents a point of reflection for both scientific research on psychedelics and empathogens and for psychoanalytic clinical practices. To date, studies on obsessive–compulsive symptomatology have primarily concentrated on the use of psilocybin and ketamine, and only one clinical study is currently examining the efficacy of combining MDMA with CBT for OCD. Against this background, the Ygg case offers a clinically illustrative example of how a long-term psychoanalysis and MDMA-assisted sessions conducted elsewhere may intersect in the treatment of OCD. This combined approach is presented here not as a demonstration of therapeutic success, but as a hypothesis-generating clinical observation. This combined approach offers a novel therapeutic perspective, while simultaneously generating hypotheses for future empirical research. From a methodological perspective, the mechanisms of action delineated in this study are not derived from standardized process measures, but from a close examination of the clinical material pertaining to a single psychoanalytic case. In keeping with psychoanalytic case study methodology, the present report is conceived not as a validation of causal efficacy, but as a paradigm that facilitates a detailed elucidation of transformative processes. Inferences regarding underlying mechanisms are thus based on clinical indicators observed within the dynamics and economy of psychoanalytic work, including changes in affective tolerance and symbolization, obsessive symptoms and relational patterns, as well as transformations within the transference–countertransference field.

To maintain a clear link between the clinical material and the broader theoretical reflections, the present discussion is organized around three interrelated analytic themes that emerged in Ygg’s treatment : (1) changes in affect regulation and emotional processing; (2) transformations in transference and the analytic field; and (3) the reorganization of traumatic memory traces, considered in dialogue with contemporary models of psychedelic-assisted therapy. Theoretical references are therefore used selectively and only insofar as they clarify these themes in the Ygg case, rather than functioning as parallel commentary.

With regard to the first theme – changes in affect regulation and emotional processing –, the distinctive states of consciousness induced by MDMA may, in certain cases, allow patients to access unconscious material in a more direct and intensified manner. Such access may contribute to the acceleration of the analytic work and facilitate movement beyond therapeutic impasses (Ringstrom, 2022). Traditionally, psychoanalysis has focused on exploring unconscious processes through verbal communication. By contrast, psychedelics and MDMA catalyze perceptual and affective shifts that may enable patients to confront and integrate buried emotional experiences and traumatic material, processes that are often crucial for therapeutic progress. These experiences tend to be more regressive and immersive than those typically encountered in ordinary psychotherapy, making it essential for the therapist to contain and manage such intensity in order to support integration rather than decompensation.

With regard to the second theme – trauma and memory – the first MDMA session brought to the surface clusters of autobiographical memories related to separation and death with an intensity that had not been achievable through years of prior analytic work. The patient was able to remain emotionally engaged with this material, to experience grief and pain in the presence of the therapists, and subsequently to revisit each scene in later analytic sessions with progressively diminishing distress and an enhanced capacity for reflection. From the vantage point of current models of MDMA-assisted psychotherapy, these observed changes – namely, greater access to distressing memories accompanied by reduced avoidance, increased emotional engagement, and the progressive re-narration of events – are consistent with the hypothesis that MDMA can serve as a catalyst for emotional processing through its effects on memory reconsolidation and fear extinction, as proposed in models of MDMA-assisted therapy for PTSD and social anxiety (Feduccia and Mithoefer, 2018; Luoma et al., 2021). Our clinical material cannot demonstrate these mechanisms; it can only be said to be phenomenologically compatible with them. Psychoanalytically, these effects may be understood as a temporary loosening of the obsessional defensive organization, allowing archaic fantasies of abandonment and neglect – previously organized defensively, within the obsessive structure and dramatized, for example, in the image of the child in uniform from the first MDMA session – to become thinkable and symbolizable.

From a psychoanalytic perspective, a second group of mechanisms concerns the third theme: transformations in the therapeutic relationship and the analytic field. Following the MDMA sessions, the patient demonstrated an increased capacity for self-reflection, a more direct expression of hatred, and greater engagement in relationships and experiences without resorting to elaborate reassurance-seeking rituals. These phenomena can be interpreted as clinical indicators of a strengthened working alliance and an expanded holding environment, with models that conceptualize MDMA-assisted therapy as an experience that enhances trust, openness, and corrective relational experiences within a structured therapeutic frame (Albert and Back, 2025; Luoma et al., 2021; O’Donnell et al., 2024). Recent psychoanalytic and relational models of psychedelic treatment similarly describe psychedelic-assisted sessions as an amplified relational field, in which affects and self-states are transmitted and transformed more intensely between the patient and therapist (Lichtenstein and Hoeh, 2024; Rundel, 2022; Stänicke, 2024). Psychedelics and empathogens appear to intensify transference and countertransference dynamics within the therapeutic relationship (Maggio et al., 2023)^11^, within these psychoanalytic and relational models.

Research on non-ordinary states of consciousness induced by psychedelics and empathogens increasingly indicates a shift from purely pharmacological models toward psychological models of therapeutic action (Weiss et al., 2024). Within these frameworks, altered states of consciousness may facilitate psychological openness, insight, and the integration of transformative experiences, thereby contributing to the repair of attachment relationships and promotion of greater psychological flexibility. Accordingly, the therapeutic effects of psychedelics appear to be transdiagnostic (Kelly et al., 2021; Kočárová et al., 2021). Our case supports this perspective by foregrounding the subjective and relational dimensions of change: what proves decisive is not the molecule *per se*, but the manner in which the experience is prepared, held, and elaborated within a meaningful therapeutic frame – in this instance, a long-term psychoanalysis. Simultaneously, and in a complementary way, the psychoanalytic process already underway seems to have catalyzed the effects of MDMA, allowing for more intensive integration work that extended for several months following each session – markedly different from the three or four integration sessions typically proposed in clinical trials. In Ygg’s case, this reciprocal process coincided with an acceleration of analytic processing and the subsequent remission of obsessive symptoms. The capacity of MDMA to evoke subjective experiences analogous to those sought in traditional psychoanalysis is particularly noteworthy. This reinforces the idea that the combination of psychedelics and psychoanalysis may represent a promising frontier in the treatment of neurosis, provided it is implemented within appropriate legal and ethical frameworks.

Two tentative conclusions can be drawn from this psychoanalytic single-case clinical narrative. First, from a psychoanalytic perspective and within the author’s clinical interpretation of this case, the MDMA experience can be understood as functioning in a manner that partially resembles the interpretive work of the analyst, in that it appears to facilitate the emergence and retrieval of latent material-content that is often distorted by dreams and symptoms. Second, in this case, the archaic content recovered through this process remained closely linked to infantile sexuality, underscoring the continued relevance of psychoanalytic theory for understanding and integrating such experiences. Any links drawn here between psychoanalytic concepts, MDMA-assisted states, and contemporary psychedelic research should therefore be understood as clinical and theoretical interpretations of a psychoanalytic single-case clinical narrative, rather than as empirically established mechanisms. More generally, any references in this article to “mechanisms” or “processes of change” are intended as psychoanalytic and clinical meanings, not as experimentally established mechanisms of action.

In conclusion, the concept of “amplified psychoanalysis” is proposed as an emerging and promising area of clinical practice and research. On the basis of this psychoanalytic single-case clinical narrative, we can tentatively venture the following definition: “amplified psychoanalysis” refers to an integrative approach in which non-ordinary states of consciousness – induced, for example, by MDMA or by classic psychedelics such as LSD, psilocybin, or DMT – are deliberately embedded within an ongoing analytic treatment in order to potentiate psychoanalytic processing and to facilitate the emergence and working-through of unconscious material. In such states, phenomena described by psychoanalytic theory – regression, symbolization, identification, condensation, transference – often appear intensified, making the underlying psychic processes more visible and workable in subsequent analytic sessions. At the same time, the pre-existing analytic frame amplifies the meaning and integrative potential of the non-ordinary experience itself, so that “amplification” is understood as a bidirectional, circular process between the substance-induced states and psychoanalytic work.

While the clinical evolution observed in the Ygg case is encouraging, this field requires further research and discussion to fully understand its potential and its limitations.

A first limitation concerns the ethical status of the material on which this report is based. The MDMA sessions took place in an underground setting outside any legal or regulatory framework. Although the author had no role in organizing, prescribing, or administering MDMA, and the patient’s safety was the responsibility of a separate therapist dyad, theorizing potential clinical benefit from such experiences inevitably raises tensions for a broader clinical readership. For this reason, the present manuscript should not be read as legitimizing or normalizing unregulated MDMA use. Any future development of “amplified psychoanalysis” is explicitly envisaged only within legally regulated, ethics-approved contexts.

A second limitation of the present report concerns outcome assessment: in keeping with psychoanalytic single-case methodology, this report relies on naturalistic clinical observation and the patient’s narrative material rather than on standardized pre- and post-treatment outcome measures. Quantitative indices of symptom severity, functional impairment, or quality of life were not collected, nor were independent clinical ratings obtained. The reported disappearance of OCD symptoms and the maintenance of change at the 18-month follow-up should therefore be understood as a clinically observed course in a single case, rather than as evidence for the therapeutic efficacy of MDMA-assisted psychoanalysis for OCD. Future research should integrate process-oriented psychoanalytic data with validated outcome measures, longer follow-up periods, and larger samples. This will need to include, for example, standardized OCD outcome measures such as the Yale–Brown Obsessive–Compulsive Scale (Y-BOCS) and the Obsessive–Compulsive Inventory–Revised (OCI-R), as well as indices of functional impairment and quality of life. On this basis, we recommend designing case series and empirical studies using MDMA-assisted therapy protocols adapted to the psychoanalytic setting. Such studies should prospectively evaluate both the efficacy and safety of this approach in larger and more diverse samples, across age, gender, and diagnostic profiles, and should include extended follow-up, building on the foundation provided by existing clinical trials. In this sense, the Ygg case is proposed as a “specimen case” (Kächele et al., 2012), a case that generates hypotheses rather than providing conclusive proof, and it invites the psychoanalytic community to participate in the development of shared research protocols that bring together the psychoanalytic tradition and evidence derived from controlled empirical studies.

## Data Availability

The datasets presented in this article are not readily available because the patient has given the freedom to consult these data only to the clinician. Requests to access the datasets should be directed to filippo.dellanoce@outlook.com.

## Footnotes

1 I would like to express my deep gratitude to my Mentor Group formed during the CPTR 2023/2024 at the *California Institute of Integral Studies* (CIIS), to our mentor Brian Richards and also to Julia King, for their patient re-reading, shared reflections, and exciting discussions around the Ygg case.

2 MDMA and classic psychedelics (such as LSD, psilocybin, and DMT) are both psychoactive substances, but they differ significantly in their chemical structure, mechanisms of action, effects, and therapeutic applications (Holze et al., 2020). Classic psychedelics primarily act as agonists at the 5-HT2A receptor, leading to altered states of consciousness, visual and auditory hallucinations, and profound changes in perception and thought processes. They do not typically produce the same level of emotional closeness or sociability as MDMA.

3 https://clinicaltrials.gov/study/NCT05783817.

4 The patient has provided written informed consent for the case to be published, and numerous biographical details have been altered to maintain confidentiality.

5 Here, for the first time, we see an element that would later become central: in his mother tongue, “*si è fatto fregare*” could means either “to be had,” “to be tricked” or, literally, “to be pinched,” “to be robbed.”

6 Ironically, but also tragically, he moved to Paris to escape his obsessions, even though his first obsession (the bogeyman) had manifested itself after the first move in his life, and the obsessions after adolescence had begun after a second move.

7 The utilization of MDMA within a therapeutic milieu gives rise to a distinct set of ethical and legal complexities. In numerous countries worldwide, the molecule remains classified as a controlled substance, whose utilization is permitted exclusively within the confines of authorized clinical trials. In Switzerland, as in Australia, MDMA can be prescribed for the treatment of resistant PTSD, subject to authorisation by the competent medical authorities. Consequently, the Ygg case cannot be regarded as a model that can be directly transferred to standard clinical practice.

8 One could perhaps note the shift from the veil to the jacket, with a consequent unveiling of the identity of the hated woman. Moreover, by recognizing the jacket is not his, Ygg is perhaps beginning a work of disidentification.

9 “*She has got me*” or “*she has stolen me*.”

10 “You are taking him away from me” or “you are stealing him away from me” are analogous here. This anger may also relate to the first analyst and two other women, for whom the expression, “*l’hanno fregato*” begins to be intelligible.

11 We must not forget that, in Ygg’s case, the MDMA interacted not only with the pre-existing transference with me as Ygg’s analyst, reinforcing it and allowing the dismantling of the defenses that hindered the analytic work, but also with the two therapists who accompanied Ygg on his journey with MDMA, a transference about which we unfortunately have no information from the patient’s testimony. Future research will need to examine situations like Ygg’s in order to analyze the specifics of diversified transferences and the changes that might occur. Nor is it pointless to question the specific place of the psychedelic therapist couple in relation to the analyst or therapist with whom the patient is already engaged in clinical work.
